# Discordance in maternal and paternal genetic markers in lesser long-nosed bat *Leptonycteris yerbabuenae*, a migratory bat: recent expansion to the North and male phylopatry

**DOI:** 10.7717/peerj.12168

**Published:** 2021-09-29

**Authors:** Roberto-Emiliano Trejo-Salazar, Gabriela Castellanos-Morales, DulceCarolina Hernández-Rosales, Niza Gámez, Jaime Gasca-Pineda, Miguel Rene Morales Garza, Rodrigo Medellin, Luis E. Eguiarte

**Affiliations:** 1 Pograma de Doctorado en Ciencias Biomédicas, Instituto de Ecología, Universidad Nacional Autónoma de México, Ciudad Universitaria, Ciudad de Mexico, México; 2 Ecología Evolutiva, Instituto de Ecología, Universidad Nacional Autónoma de México, Ciudad Universitaria, Ciudad de México, México; 3 Conservación de la Biodiversidad, El Colegio de la Frontera Sur, Villahermosa, Tabasco, Mexico; 4 Facultad de Estudios Superiores Zaragoza, Universidad Nacional Autónoma de México, Ciudad de Mexico, Ciudad de Mexico, Mexico; 5 Facultad de Ciencia y Tecnología, Universidad Simón Bolívar, Ciudad de Mexico, Ciudad de Mexico, Mexico; 6 Ecología de la Biodiversidad, Instituto de Ecología, Universidad Nacional Autónoma de México, Ciudad de Mexico, Ciudad de Mexico, Mexico

**Keywords:** Migratory bat, Phylogeography, Parent lineages, Demographic expansion, Cytochrome-b

## Abstract

*Leptonycteris yerbabuenae*, the lesser long-nosed bat is an abundant migratory nectar-feeding bat found in most of Mexico, and in some areas of northern Central America and small sections of southwestern USA. We analyzed the distribution of the maternal and paternal lineages of this species with phylogeographic methods based on two mitochondrial markers, *Cyt-b* and *D-loop*, and a marker located in the Y chromosome, *DBY*. We obtained tissue samples from 220 individuals from 23 localities. Levels of genetic diversity (haplotype diversity, *H_d_*) were high (*Cyt-b* = 0.757; *D-loop* = 0.8082; *DBY* = 0.9137). No clear patterns of population genetic structure were found for mitochondrial markers, while male genetic differentiation suggested the presence of two lineages: one from Mexican Pacific coast states and another from central-southern Mexico; in accordance to strong male philopatry and higher female migration. We used genealogical reconstructions based on Bayesian tools to calculate divergence times, and to test coalescent models to explain changes in *L*. *yerbabuenae* historical demography. Our results show that recent demographic changes were consistent with global climatic changes (∼130,000 kyr ago for *Cyt-b* and ∼160,000 kyr for *D-loop*) and divergence times dated from molecular genealogies exhibited older divergence times, *Cyt-b* (4.03 mya), *D-loop* (10.26 mya) and *DBY* (12.23 mya). Accordingly, the female lineage underwent demographic expansion associated to Pleistocene climate change, whereas the male lineage remained constant.

## Introduction

Climate oscillations have global and local effects on distribution, demographic and genetic diversity patterns (Bloom et al., 2013; Ramírez-Barahona & Eguiarte, 2013; Castellanos-Morales et al., 2016; Scheinvar et al., 2017; Hipp et al., 2018). In particular, Mexico experienced several geological and climatic changes in the recent past (Pleistocene), which have promoted speciation, extinction and diversification of its flora (Ramírez-Barahona & Eguiarte, 2013; Scheinvar et al., 2017; Hipp et al., 2018) and fauna (Gutiérrez-García & Vázquez-Domínguez, 2013; Trejo-Salazar, 2013; Castellanos-Morales et al., 2016). These phylogeographic and biogeographic analyses have shown that genetic patterns of Mexican taxa have been mediated by ecological, geological and climate changes. Aspects of the natural history of species, such as annual migration, are seldom considered in the analysis of species’ ecological and evolutionary history because of its complexity, but potentially can be very important. For instance, migration can affect genetic structure, especially in cases were each sex exhibits different migration patterns during the reproductive season (Russell, Medellín & McCracken, 2005).

The lesser long-nosed bat, *Leptonycteris yerbabuenae*, (Glossophaginae) is considered the most widespread nectar-feeding bat in Mexico, pollinating at least 64 plant species, including many *Agave* species, columnar cacti and Bombacoideae trees (Rojas-Martínez et al., 1999; Fleming, Geiselman & Kress, 2009; Trejo-Salazar, Scheinvar & Eguiarte, 2015; Bustamante et al., 2016; Jiménez-Barrón et al., 2020). Distribution of *L. yerbabuenae* comprises mainly arid and semiarid areas (Cole & Wilson, 2006) from Nicaragua to Arizona, including most of Mexico, except for the Yucatan peninsula and north of Baja California, there are no records of its presence in the coast of the Gulf of Mexico and in the northern area of the Chihuahuan desert (Cole & Wilson, 2006; Tapia, Ñamendy & Martínez-Fonseca, 2020). It is listed as Near Threatened with decreasing population trends (IUCN Red List of Threatened Species, 2017), but was delisted in Mexico since the populations have recovered (Medellín et al., 2018).

The lesser-long nosed bat is a migratory species (Cole & Wilson, 2006). The migration involves only female groups that travel north from central and southern Mexico during spring-summer to give birth (Arizona and Sonoran Desert), and they travel back south in autumn (Wilkinson & Fleming, 1996; Ceballos et al., 1997; Rojas-Martínez et al., 1999; Medellín et al., 2018). This migration is associated with the blooming period of cactus during summer at the northern area (Fleming, Nuñez & Sternberg, 1993; Molina-Freaner & Eguiarte, 2003; Burke et al., 2019) and of agaves during the journey (Burke et al., 2019). In addition, all the males and some females remain in permanent roost caves in the center-south of its distribution area year-round (Rojas-Martínez et al., 1999; Riechers, Martínez-Coronel & Vidal, 2003; Stoner et al., 2003; Galindo et al., 2004). This raises questions regarding the proportion of the female population that migrates, the magnitude of the sex ratio bias in permanent locations at given times due to female migration, and the identity and steadiness of migrant females. Also, we could expect to find differences in the population genetic structure of locations where the species is present throughout the year.

Previous genetic studies based on mitochondrial, microsatellites and Random Amplified Polymorphic DNA markers (RAPDs) have concluded that there are different genetic groups along the species distribution (Wilkinson & Fleming, 1996; Morales-Garza et al., 2007). Wilkinson & Fleming (1996) proposed a coastal and an inland group, in accordance with migration routes, supported by Rojas-Martínez et al. (1999) and Menchaca et al. (2020). However, these studies did not analyze resident groups from permanent roosting caves. Morales-Garza et al. (2007) found that the species is composed by at least a “center-south” and a “west-north” group; but their samples only covered six sites (Baja California, Sonora, Hidalgo, Morelos, Puebla and Oaxaca); while Arteaga et al. (2018) reported a demographic expansion in Baja California. These studies provide evidence for the existence of at least two genetic groups, and here we analyze if these genetic groups could relate to differences in migration behavior between males and females in *L. yerbabuenae*.

**Figure 1:**
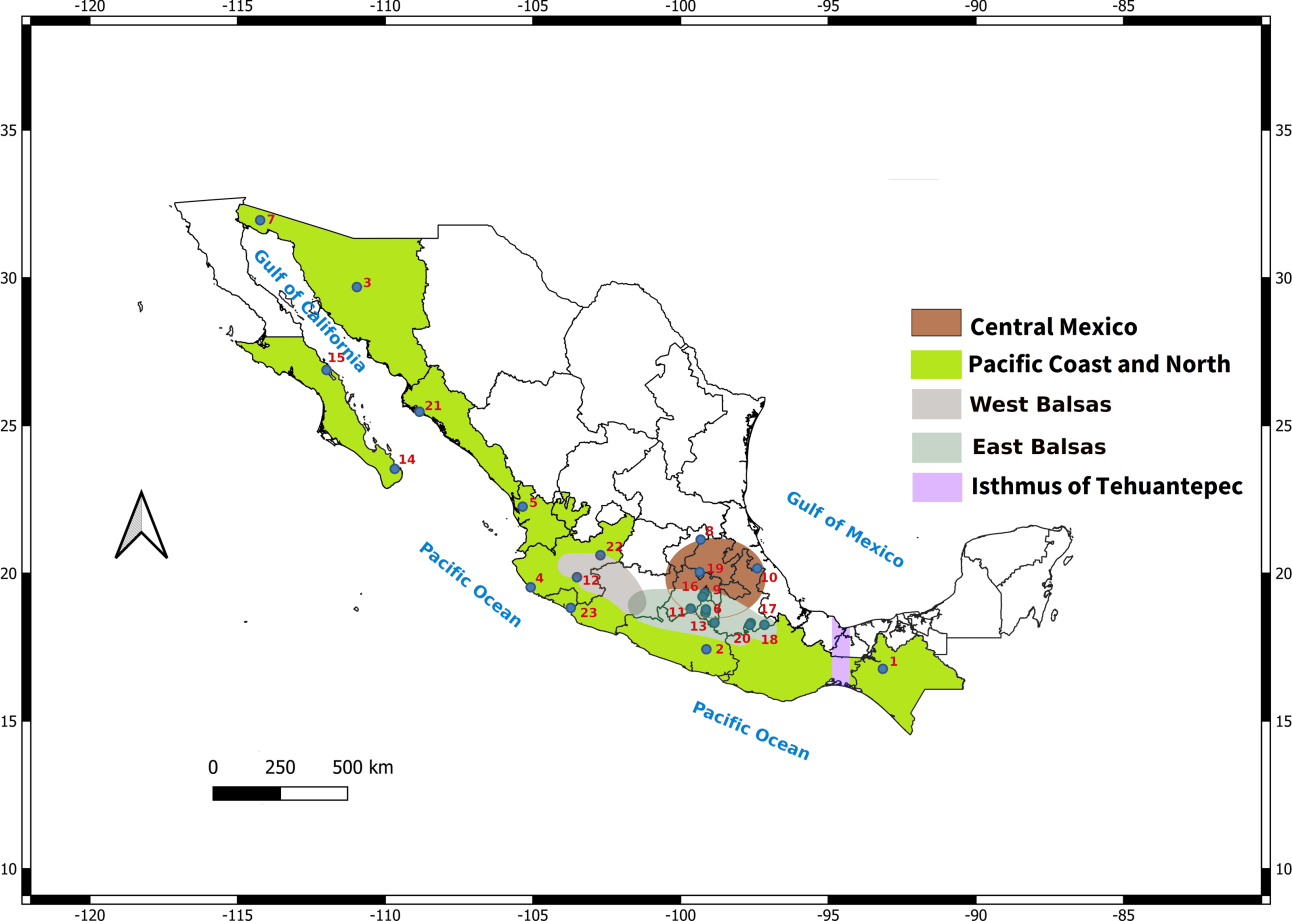
Map depicting sampled localities showing the Balsas River Basin and Isthmus of Tehuantepec. The Balsas River divides the Central Mexico-East Balsas and West Balsas-Pacific Coast groups proposed by Morales-Garza et al. (2007).

In addition, the distribution of *L. yerbabuena* encompasses several biogeographic regions (Fig. 1), that have influenced the distribution of genetic variation in some bat species. For example, the Isthmus of Tehuantepec is a genetic barrier for *Pteronotus davyi* (Guevara-Chumacero et al., 2010), *P. personatus* (Zárate-Martínez et al., 2018) and *Natalus mexicanus* (López-Wilchis et al., 2021), while *Artibeus jamaicensis* show the presence of two lineages: Gulf of Mexico and Pacific Ocean (Ruiz, Vargas-Miranda & Zúñiga, 2013). The region around the Balsas river is also an important barrier for species such as *Sturnira parvidens* (Hernández-Canchola & León-Paniagua, 2017). In these studies, geographical barriers together with Pleistocene climate changes promoted lineage divergence followed by range expansion (Guevara-Chumacero et al., 2010; Ruiz, Vargas-Miranda & Zúñiga, 2013; Hernández-Canchola & León-Paniagua, 2017; Zárate-Martínez et al., 2018; López-Wilchis et al., 2021).

In the present study, we analyzed paternally and maternally inherited molecular markers to uncover the effect of sex-bias in migration patterns on the distribution of genetic diversity in *L. yerbabuenae*. We conducted a phylogeographic analysis with a sampling covering the species distribution in Mexico, and test whether climatic changes have influenced its demographic dynamics. We hypothesize that past global climate change had a differential effect in the genetic diversity, phylogeographic patterns and demographic history of female and male lineages of *L. yerbabuenae*, and migratory movements could be the result of a geographic expansion related to changes that represent better climatic and ecological conditions for this species in the present.

## Materials & Methods

### Sampling

**Figure 2:**
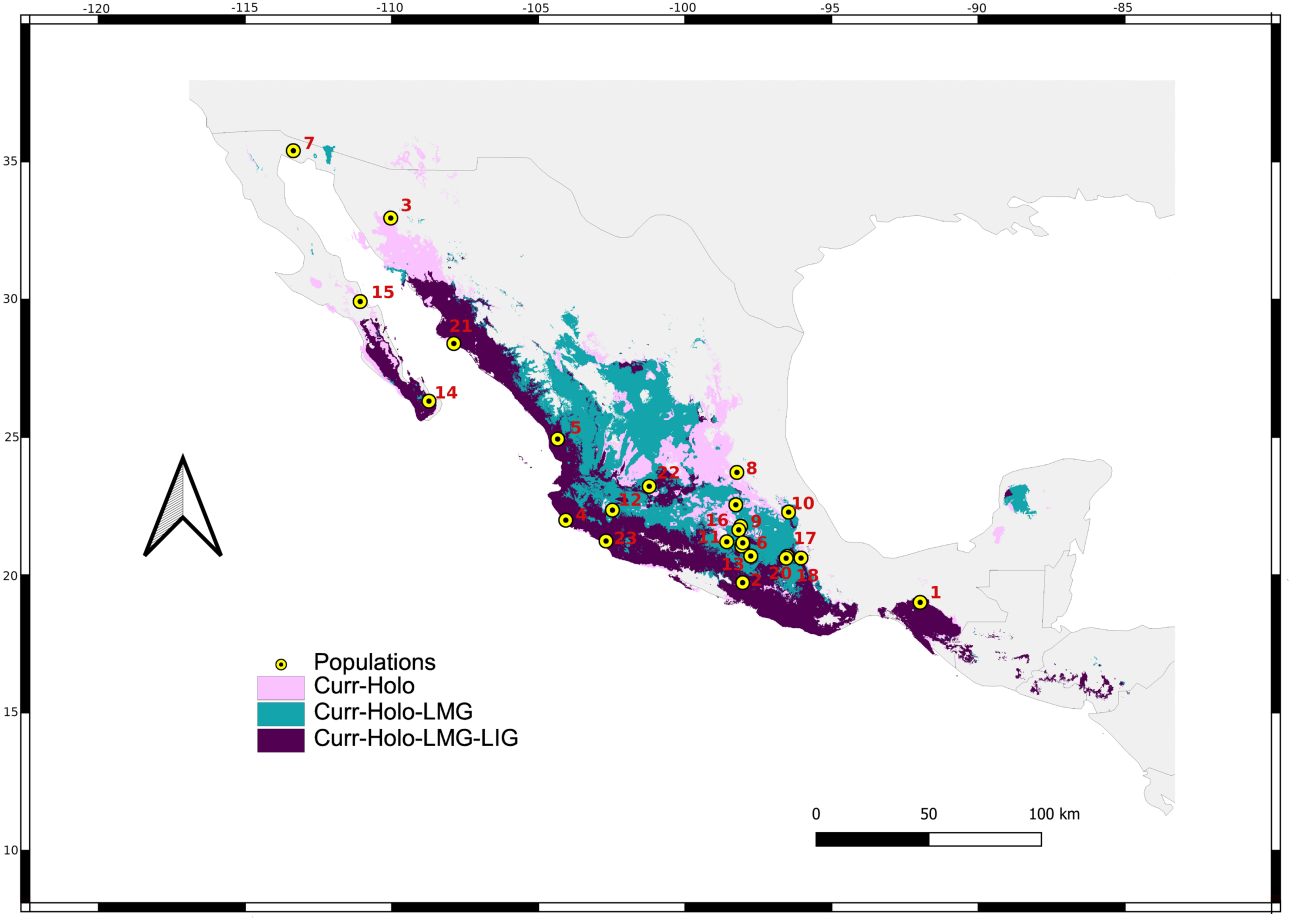
Sum of maps. Map depicting species distribution models past projections highlighting areas of environmental stability into the current-Holocene (pink), current-Holocene-Last Glacial Maximum (blue), and current-Holocene-Last Glacial Maximum-Last Interglacial (purple). Yellow points show sampled localities.

Samples were taken from 23 localities in roosting caves and mist nest on field feeding-sites along *L. yerbabuenae* distribution from 2014 to 2016 (Fig. 2), with sampling permit Secretaría del Medio Ambiente y Recursos Naturales (SEMARNAT) SGPA/DGVS/07161/15 (Supplemental File S1) following the Animal Care and Use protocols of the American Society of Mammalogists (Sikes, 2016). Tissue samples were taken with a three mm^2^ biopsy wing punch in an area of the wing with no blood capillaries or nerve terminals. Wing biopsies were fixed in 90% ethanol at environmental temperature and then stored at −20 °C until DNA extraction. Additionally, we used tissue samples from wing punches collected from 2001 to 2003 (Morales-Garza et al., 2007) and tissue samples from wing punches from the tissue collection of the Laboratorio de Ecología y Conservación de Vertebrados Terrestres (LECVT), Instituto de Ecología, UNAM (Table 1).

**Table 1:** Number of male and female samples for *Leptonycteris yerbabuenae* sequenced for *Cytb*, *D-loop* and *DBY* regions (only males) for each Locality and State. Locality ID key is shown Next to locality.

		*Cyt-b*			*D-loop*			*DBY*	Geographic coordinates	
Locality		Male	Female	**Total**	Male	Female	**Total**	*n*	Lat	Long
1 Los Laguitos, Chiapas	Chi	10	1	**11**	1	10	**11**	1	−93.15098	16.76512
2 Juxtlahuaca, Guerrero	Jux	3	21	**24**	3	22	**25**	9	−99.12634	17.42676
3 La Mariana, Sonora	Son	6	12	**18**	2	14	**16**	1	−110.9587	29.6864
4 Chamela, Jalisco	Chame	11	4	**15**	1	0	**1**	19	−105.0738	19.52677
5 Las Lumbres, Nayarit^*^	Nay	0	1	**1**	0	0	**0**	8	−105.3465	22.25478
6 Ticuman, Morelos^*^	Tic	0	10	**10**	0	7	**7**	0	−99.14083	18.77555
7 El Pinacate, Sonora	Pin	0	20	**20**	0	18	**18**	0	−114.2341	31.94816
8 Xoxafi, Hidalgo^*^	Xox	1	0	**1**	0	1	**1**	2	−99.319	21.137
9 Las Vegas, Puebla^*^	vegas	0	5	**5**	0	0	**0**	0	−97.40263	20.16594
10 Tzinacanostoc, Puebla^*^	Tzni	0	1	**1**	0	0	**0**	0	−98.85219	18.32304
11 Tonatico, Estado de México	Ton	2	2	**4**	0	0	**0**	2	−99.65836	18.80343
12 Atotonilco, Jalisco	Ato	4	3	**7**	4	2	**6**	10	−102.7132	20.61148
13 Tetecalita, Morelos (Salitre)	Sal	16	10	**26**	12	8	**20**	21	−99.15740	18.65918
14 Baja California (Las Cuevas)	BC1	2	7	**9**	1	3	**4**	6	−109.6773	23.532
15 Baja California (Mulege)	BC2	8	1	**9**	0	0	**0**	6	−111.9868	26.8785
16 Ciudad de México	DF	2	9	**11**	9	0	**9**	9	−99.19241	19.32163
17 San Juan Raya, Oaxaca	SJR	23	9	**32**	21	6	**27**	16	−97.62269	18.31159
18 San Sebastian Frontera, Oax	SSF	4	1	**5**	3	0	**3**	0	−97.65934	18.24915
19 Tula, Hidalgo	Tul	1	2	**3**	3	0	**3**	1	−99.35589	20.04357
20 Coxcatlán, Oaxaca	Cox	0	1	**1**	0	1	**1**	0	−97.16104	18.25364
21 Navachiste, Sinaloa^*^	Sin	0	0	**0**	0	0	**0**	1	−108.8382	25.46586
22 Tuxtepec, Jalisco^*^	Tux	0	0	**0**	0	0	**0**	11	−103.5061	19.8661
23 Callejones, Colima^*^	Col	0	0	**0**	0	0	**0**	7	−103.7177	18.82904
	** **			**213**			**152**	**130**		

**Notes.**

* Samples from LECTV historical tissue collection.

### DNA extraction and amplification

Total genomic DNA was extracted following Paboö’s modified protocol (Gasca Pineda, 2015). Tissue was digested for 12 h at 40 °C in Paboö lysis solution (100 mM NaCl, 100mM Tris HCl and 2mM EDTA, pH8.0) with 20 mg/ml proteinase K, 2% SDS and 0.04M DTT, followed by a phenol: chloroform protocol for DNA isolation (Gasca Pineda, 2015). The quality and amount of extracted DNA was visualized in a 1% agarose gel. We performed gel electrophoresis at 90 V for 30 min; gel was stained with Midori green advance solution and visualized in UV light. We sequenced two mitochondrial DNA regions, cytochrome b (*Cyt-b*) and control region (*D-loop*), which are maternally inherited and variable in most mammal species. We also sequenced the region of Dead box associated to the Y chromosome gene (*DBY*) which is located in the Y chromosome and is paternally inherited, this marker has been used before for phylogeographic studies in phyllostomid bats (Clare et al., 2011).

We obtained 1121 bp of *Cyt-b* with primers L14125 5′TGAAAAAYCATCGTTGT 3′and H15915 5′TCTTCATTTYWGGTTTACAAGAC 3′(Steppan et al., 1999). For the *D-loop* region , we amplified 828 bp with primers L15933 5′-CTCTGGTCTTGTAAACCAAAAATG-3′and H637 5′-AGGACCAAACCTTTGTGTTTATG-3′(Oshida et al., 2001). PCR for mitochondrial markers were performed in a final total reaction volume of 15 µl, and contained 2 µl of DNA, 2 U of Taq polymerase (GoTaq Flexi DNA Polymerasa, Promega, USA), 0.4 µM of each primer (10 µM), 1x Taq buffer, 2.5 µM of MgCl_2_ (25 µM), 0.2 µM of dNTPs (10 µM) and 7.325µl of H_2_O. The PCR profile for *Cyt-b* was: 5 min of initial denaturation at 95 °C, followed by 35 cycles of 30 s at 96 °C, 1 min at 53 °C, 2 min at 72 °C, and a final extension of 7 min at 72 °C; and for *D-loop*: 3 min at 95 °C, followed by 35 cycles of 30 s at 94 °C, 45 s at 54 °C, 2 min of 72 °C, and a final extension of 10 min at 72 °C in an ABI Veriti 96-Well Thermal Cycler (Model: 9902; Thermo Fisher Scientific Inc.).

We amplified a 450 bp fragment from the Dead box associated to the Y chromosome gene (*DBY*) with primers 5′-CCGTTACTTCCATTTTCAAAA-3′and 5′-GCTAAAACCAACGAGATTGGT-3′(Lim, 2007; Lim et al., 2008); the reaction mixture of 15µl total volume contained 2µl of genomic DNA, 2 µM of each primer (10 µM), 200 µM dNTPs (10 µM), 1.5 mM MgCl_2_ (25 µM), 2.5 U of Taq DNA polymerase (Promega) and 7.7µl of H_2_O. Amplification was carried out as follows: 10 min of an initial denaturation at 94 °C, 36 cycles of 45 s at 94 °C, 30 s at 54 °C, 2:30 min at 72 °C, and a final extension of 5 min at 72 °C in an ABI Veriti 96-Well Thermal Cycler (Model: 9902; Thermo Fisher Scientific Inc.). We sequenced each genetic region with forward and reverse primers at Macrogen USA’s Maryland headquarters (http://www.macrogenusa.com).

Differences in the final sample number among markers resulted from PCR artifacts, *Cyt-b* was successfully amplified, while for *D-loop* sequences were low quality for several individuals and were discarded from analyses. We amplified a total of 213 individuals for *Cyt-b*, 152 for *D-loop* and 130 for *DBY* (Table 1; Supplemental File S2). All sequences are available in NCBI GenBank (accession number: *Dloop*: MT790834–MT790986; *Cytb*: MT859334–MT859403; *DBY*: MT913638–MT913767).

### Data analysis

#### Genetic diversity

We assessed the quality of DNA sequences and assembled forward and reverse sequences with Consed 29.0 using the default settings (Ewing et al., 1998; Gordon, 2004). We aligned sequences with CLUSTAL X (Thompson et al., 1998), and checked the alignment by hand. Missing data or undetermined bases were excluded from analyses, and we eliminated sequences with more than 50% of missing data.

For each marker, we estimated the number of segregating sites (S), number of haplotypes (h), haplotype diversity (*H*_*d*_) and nucleotide diversity (*π*) for each sampled locality using DNAsp 5.10.01 (Librado & Rozas, 2009) and Arlequin 3.5.1.2 (Excoffier & Lischer, 2010).

#### Population genetic structure

To visualize the genealogical relationship among haplotypes, we obtained a haplotype network for each molecular marker. Haplotype networks were constructed with the median-joining algorithm (Bandelt, Forster & Röhl, 1999) implemented in the program PopART 1.7 (Leigh & Bryant, 2015a; Leigh & Bryant, 2015b).

To assess genetic differentiation among populations, we calculated pairwise *F*_*ST*_ values using the Slatkin method (Slatkin & Hudson, 1991) implemented in Arlequin 3.5.1.2 (Excoffier & Lischer, 2010). We also conducted a Bayesian analysis of population structure, BAPS 6 (Corander, Sirén & Arjas, 2008). BAPS 6 uses maximum likelihood and MCMC under the non-reversible Metropolis-Hasting method to assign individuals to different genetic groups, where the number of groups is given under the assumption of admixia (Corander, Sirén & Arjas, 2008). Moreover, BAPS 6 can incorporate geographical coordinates and perform Voronoi’s tessellation to explicitly test for spatial genetic structure (Corander, Sirén & Arjas, 2008). To test whether we could recover a Pacific-South and Central Mexico groups, following Morales-Garza et al. (2007), we conducted the BAPS 6 analysis without including the geographical information and using a different fixed value of K = 2, 3, 4 and 5 and 10 repetitions for each value. To evaluate the partitioning of genetic variation among sampled localities, we performed an analysis of molecular variance (AMOVA) with poppr (Kamvar, Tabima & Grünwald, 2014) in R 1.4.1106 (R Core Team, 2013). All samples were treated as a single group to determine the amount of variation partitioned among and within the localities. To assess the significance of the two geographic groups previously analyzed with BAPS 6, we conducted an additional AMOVA. We obtained the level of significance for both tests with 10,000 permutations.

### Historical analyses

#### Divergence times

For divergence time estimations, we used the haplotype identities for each gene (*Cyt-b* (*n* = 76 haplotype sequences), *D-loop* (*n* = 43) and *DBY* (*n* = 70). We conducted separate analyses for each gene, because each region shows a different mutation rate; thus, allowing to make inferences at different time scales and for each sex. We determined the substitution model with best fit to our data with jModelTest 2 (Posada, 2008) based on the Akaike Information Criterion (AIC; Akaike, 1974). *Cyt-b* and *D-loop* followed a GTR+G+I model with *γ*-distributed rate heterogeneity, while *DBY* followed a GTR+G substitutions model.

For each region, we obtained an ultrametric tree and estimated divergence times under a relaxed uncorrelated lognormal clock model with BEAST 1.10.4 (Suchard et al., 2018), which allows rates to vary among branches. We downloaded from GenBank sequences for each region to be used as outgroups; nevertheless, we had to use different species as outgroups for each analysis in accordance with the available data. For *Cyt-b* we considered *Glossophaga commisarissi* (GenBank accession number: AF382886; Hoffmann & Baker, 2001) and *Choeronycteris mexicana* (Flores-Abreu et al., 2019) as outgroups. For *D-loop*, outgroups were *Hylonycteris underwoodii* (GenBank accession number: MF804191.1; Cruz-Salazar et al., 2018) and *Glossophaga longirostris* (GenBank accession number: AF510544.1; Newton, Nassar & Fleming, 2003). Outgroups for the *DBY* genealogy were *Glossophaga soricina*, *Uroderma bilobatum* and *Platyrrhinus helleri* (GenBank accession numbers: JF458413.1, JF458602.1 and JF458470.1, respectively; Clare et al., 2011). Genealogies were calibrated using the same four dates. Two calibration points came from the fossil record: glossophaginae 22.8 million years ago (mya) (1.5 Standard Deviation, SD) (Teeling, 2005); Choeonycterinii 13 mya (1.0 SD) (Czaplewski et al., 2003); Calibration points for *Glossophaga* + *Leptonycteris* clade15 mya (1.0 SD), and *Leptonycteris* 12 mya (1.0 SD) were derived from detailed Bayesian analysis previously reported (Flores-Abreu et al., 2019). Priors for BEAST 1.10.4 were set with the default values, running for 500 million generations sampling every 1000 generations, and a 10% burn in. We used Tracer 1.7.1 (Rambaut et al., 2018) to evaluate convergence and stationarity of 10,000 trees. The maximum credibility tree was obtained with TreeAnnotator 1.10.4 (Suchard et al., 2018) and visualized with FigTree 1.4 (Rambaut & Drummond, 2007).

### Historical demography analysis

To estimate the demographic dynamics of *L. yerbabuenae* through time we constructed Bayesian skyline plots with BEAST 1.10.4 (Suchard et al., 2018). We calculated coalescence times for each locus separately considering all individuals (*Cyt-b n* = 213, *D-loop n* = 156 and *DBY n* = 130) using GTR+G+I in a Piecewise-linear nucleotide substitution model (Suchard et al., 2018).

Genealogies and model parameters for each lineage were sampled every 50,000 iterations for 5 × 10^8^ generations under a relaxed lognormal molecular clock with uniformly distributed priors and a pre-burn in of 1000. Demographic plots for each analysis were visualized with Tracer 1.7.1 (Rambaut et al., 2018). To scale the time of the Bayesian coalescence of the Skyline (evolutionary time between real time), we used the last divergence time of the branches in the calibrated tree. We also calculated Tajima’s *D* (Tajima, 1989) and Fu’s *Fs* (Fu, 1997) with a coalescent algorithm in DNAsp 5.10.01 (Librado & Rozas, 2009), as an independent test for demographic inferences and to provide further support to skyline plot results.

#### Past distribution models

We obtained coordinates for the presence of *L. yerbabuenae* from scientific collections (Colección Nacional de Mamíferos (Instituto de Biología, UNAM), Museo de Zoología “Alfonso L. Herrera” (Facultad de Ciencias, UNAM), Colección de Mamíferos UAM-I (Universidad Autónoma Metropolitana), Colección Mastozoológica ENCB (Instituto Politécnico Nacional)) and GBIF (GBIF.org, 2014) databases and own collections (Supplemental File S3). To avoid spatial autocorrelation and bias in the distribution model estimation, we eliminated duplicate records by pixel and records separated by less than 1 km. An additional validation was carried out using information on habitat, distribution, and taxonomic status.

We used 19 bioclimatic variables (WorldClim, http://www.worldclim.org) to perform a variance inflation factor analysis to eliminate correlated bioclimatic variables, leaving a set of uncorrelated informative variables to be used in environmental niche modeling, and to obtain Mahalanobis distances. The later was used along with occurrence data to identify outlier records that could be a source of error. These points were removed from further analyses. All analyses were performed with R 1.4.1106 (R Core Team, 2013).

Climatic Niche Models were constructed with MaxEnt 3.3 (Phillips & Dudík, 2008), using the *ad-hoc* selection of variables to reduce overprojection (Hijmans et al., 2005). We executed MaxEnt 3.3 using the following settings: 20% random test, 30 bootstrap replicates, 1000 maximum iterations, convergence threshold of 0.00001, with extrapolation and clamping turned off. For the purposes of this study, we derived the distributional model for *L. yerbabuenae* from the average model. We evaluated all the distributional models using the area under the receiver operating characteristic curve (AUC) scores, where values above 0.85 are considered useful (Elith et al., 2006).

Climatic niche models (CNM) for *L. yerbabuenae* were obtained with MaxEnt 3.3 (Phillips, Dudík & Schapire, 2004; Phillips, Anderson & Schapire, 2006; Phillips & Dudík, 2008) for four periods: Current, Holocene (Hol ∼6,000 years ago), Last Glacial Maximum (LGM ∼21,000 years ago) and Last Interglacial (LIG ∼120,000–140,000 years ago). We loaded the corresponding past layers of Atmospheric-Ocean General Climate Model (AOGCM), obtained from WorldClim 1.4 dataset (Hijmans et al., 2005). The data of these layers were based on the AOGCM of the Community Climate System Model (CCSM; Collins et al., 2006). A binary map was created using the percentile value of training sample points as a threshold, assuming that 10% of records used for model generation are susceptible to error. Finally, we built a sum of maps that reflects the most stable climatic zones through these four time periods.

## Results

### Genetic diversity

We analyzed a total of 250 individuals from 23 sites across Mexico (females = 120, males = 130) (Table 1; Fig. 1). We obtained a total of 1,128 bp for *Cyt-b* (*n* = 213; 76 haplotypes); 828 bp for *D-loop* (*n* = 152; 43 haplotypes) and 450 bp for *DBY* (*n* = 130; 70 haplotypes). Genetic diversity (*H*_*d*_) values were lower for maternally inherited mitochondrial regions (*Cyt-b* = 0.757; *D-loop* = 0.8082) than for paternally inherited *DBY* (0.91; Table 2), a similar pattern was observed for nucleotide diversity (Table 2).

### Genetic structure

**Figure 3:**
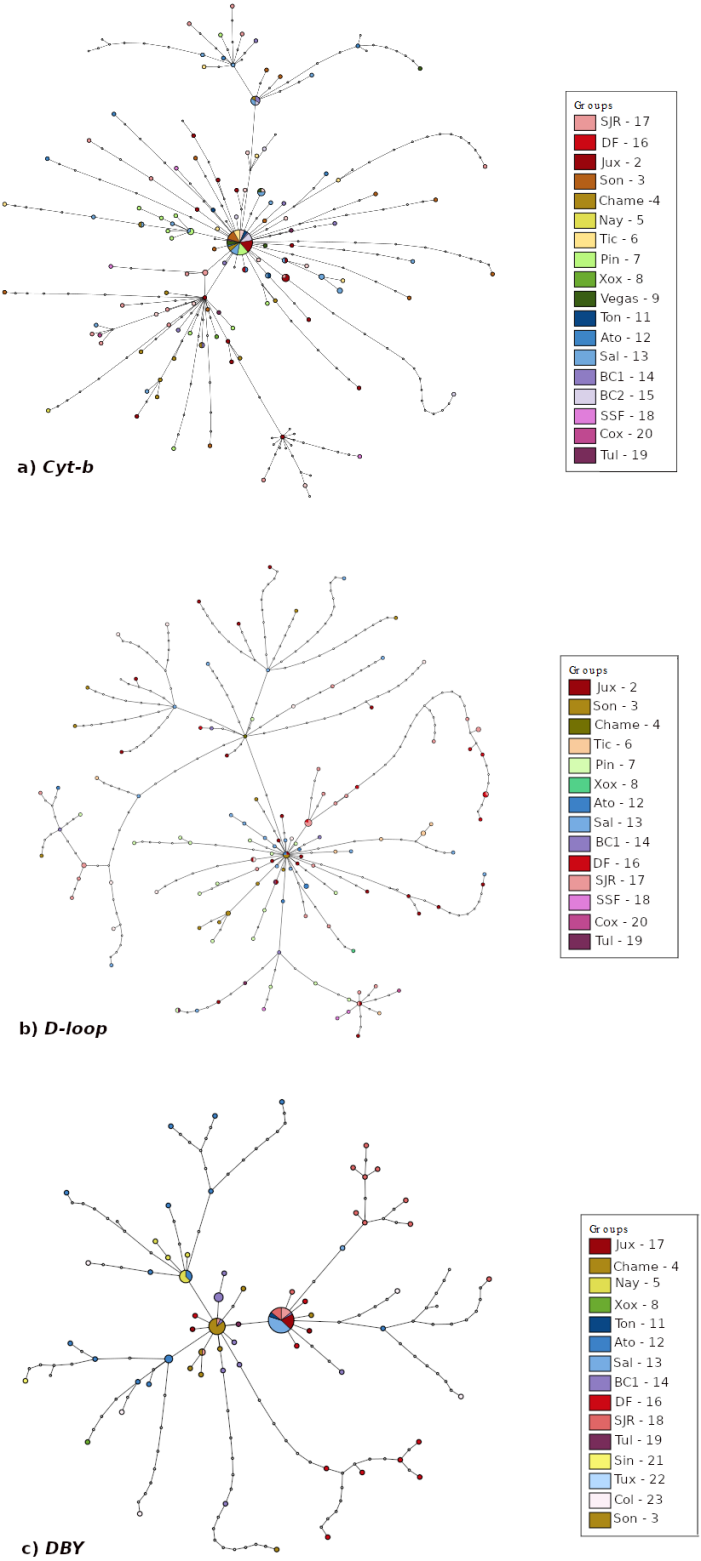
Median joining haplotype network built based on (A) *Cyt-b*, (B) *D-loop*, and (C) *DBY* markers for *Leptonycteris yerbabuenae*.

The haplotype networks (Fig. 3) exhibited differences among them. Mitochondrial *Cyt-b* and *D-loop* networks showed a main group with most haplotypes from all localities connected, and smaller groups consisting mainly of members from the Peninsula of Baja California-West of Balsas localities (Figs. 3A–3B). The *DBY* haplotype network consisted of three-star groups with geographic congruence (West of Balsas, Central Mexico and Isthmus of Tehuantepec) connected by several mutational steps (Fig. 3C).

**Table 2:** Values of genetic diversity for mitochondrial DNA *Cyt-b* and *D-loop* and for the Y-chromosome *DBY* gene showing total number of amplified individuals (n), number of segregating sites (S), haplotype number (h), haplotype diversity (Hd), and nucleotide diversity (*π*).

	Sample size (*n*)	Segregating sites (S)	Number of haplotypes (h)	Haplotype diversity (*H*_*d*_)	Nucleotide diversity (*π*)
*Cyt-b*	213	182	74	0.757	0.03087
*D-loop*	152	327	43	0.8082	0.04289
*DBY*	130	178	70	0.9137	0.04781

Pairwise *F*_*ST*_ showed low genetic differentiation between localities for the mitochondrial markers (Tables S1–S2) but higher genetic differentiation for *DBY* (Table S3). For *Cyt-b* and *D-loop*, the results suggest that locations from the West of Balsas region are moderately differentiated from East of Balsas, with values below *F*_*ST*_ = 0.4. In contrast, the paternal lineage (*DBY* marker) exhibited a well-defined genetic structure between the Isthmus of Tehuantepec-East of Balsas and West of Balsas (Pacific Coast) with values ranging from 0.6 to 0.98.

Bayesian population analysis (BAPS) for *Cyt-b* (log likelihood = −10512.121) and *D-loop* (log likelihood = −10990.658) detected two genetic clusters (*K* = 2; Fig. S1), consistent with East of Balsas *vs.* West of Balsas (or Pacific Coast) groups. In the case of the *DBY* gen, BAPS detected three different genetic groups, congruent with the geographic distribution of sampled localities (*K* = 3; log likelihood = −6397.346; S2. Fig. S1), corresponding with West of Balsas, Central Mexico, and Isthmus of Tehuantepec zones (Fig. 1).

AMOVA analyses revealed that variance was better explained without considering a hierarchical grouping for both mtDNA regions, while for *DBY* clustering provided a slightly better explanation of genetic variance (Table 3). In all cases, most of the genetic variation was found within-localities (55.2% for *Cyt-b*, 75.7% for *D-loop* and 66.6% for *DBY*) (Table 3).

**Table 3:** Results from analysis of molecular variance (AMOVA) for samples from *Leptonycteris yerbabuenae* considering: (A) All populations as a single cluster and (B) two geographic regions of the Balsas River Basin as proposed by Morales-Garza et al.,

Analysis	Source of variation	df	percentage of variation		*F*-statistic			
***Cyt-b***						
A) All populations	Among populations	21	44.74		
	Within populations	191	55.26		*F*_*ST*_= 0.447			
	Total	212		
B) Clusters				
	Among Cluster	21	21.32		
	Within clusters	13	16.25		
	Within samples	178	50.33		*F*_*CT*_= 0.334			
					*F*_*SC*_= 0.244			
	Total	212			*F*_*ST*_= 0.497			
***D-loop***						
A) All populations	Among populations	14	24.25					
	Within populations	137	75.75		*F*_*ST*_= 0.242			
	Total	151						
B) Clusters								
	Among Cluster	1	5.31					
	Within clusters	2	5.71					
	Within samples	148	88.98		*F*_*CT*_= 0.11			
					*F*_*SC*_= 0.06			
	Total	151			*F*_*ST*_= 0.053			
***DBY***				
A) All populations	Among populations	20	33.37					
	Within populations	104	66.63		*F*_*ST*_= 0.334			
	Total	124						
B) Clusters								
	Among Cluster	1	9.96					
	Within clusters	123	90.04		*F*_*CT*_= 0.344			
					*F*_*SC*_= 0.321			
	Total	124			*F*_*ST*_= 0.345			

### Historical analyses

### Divergence times

**Figure 4:**
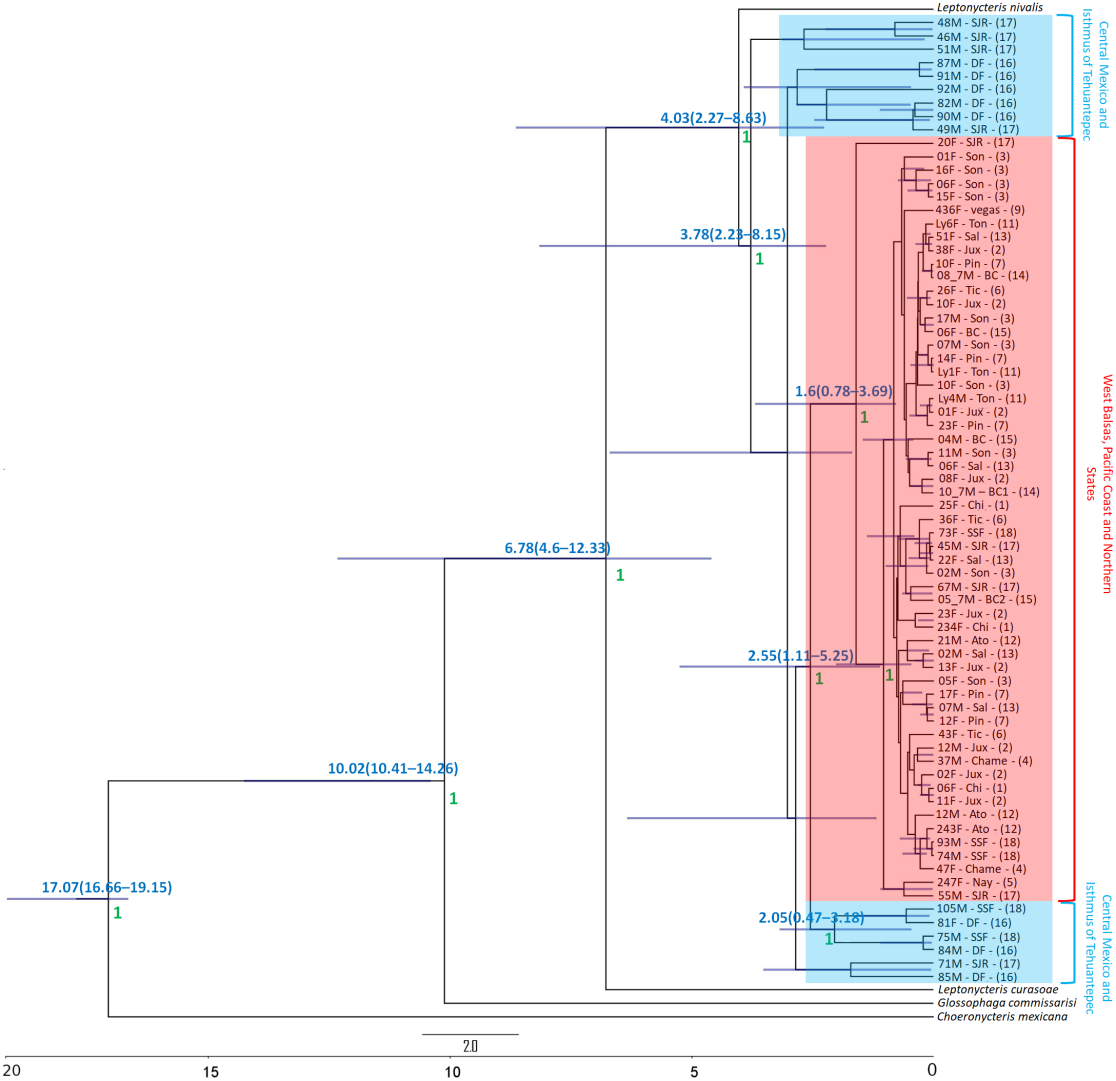
Gene genealogy and dates of divergence estimated with BEAST based on paternally inherited *Cyt-b* sequences. Black numbers above nodes depict divergence in million years; green numbers below nodes depict support values (posterior probability). Highlighted in blue color are the individuals from the Central Mexico and Isthmus of Tehuantepec localities, highlighted in red color are the individuals from the localities located in the West Balsas-Pacific Coast and Northern States Region. HDP values are in parentheses above the blue bar.

**Figure 5:**
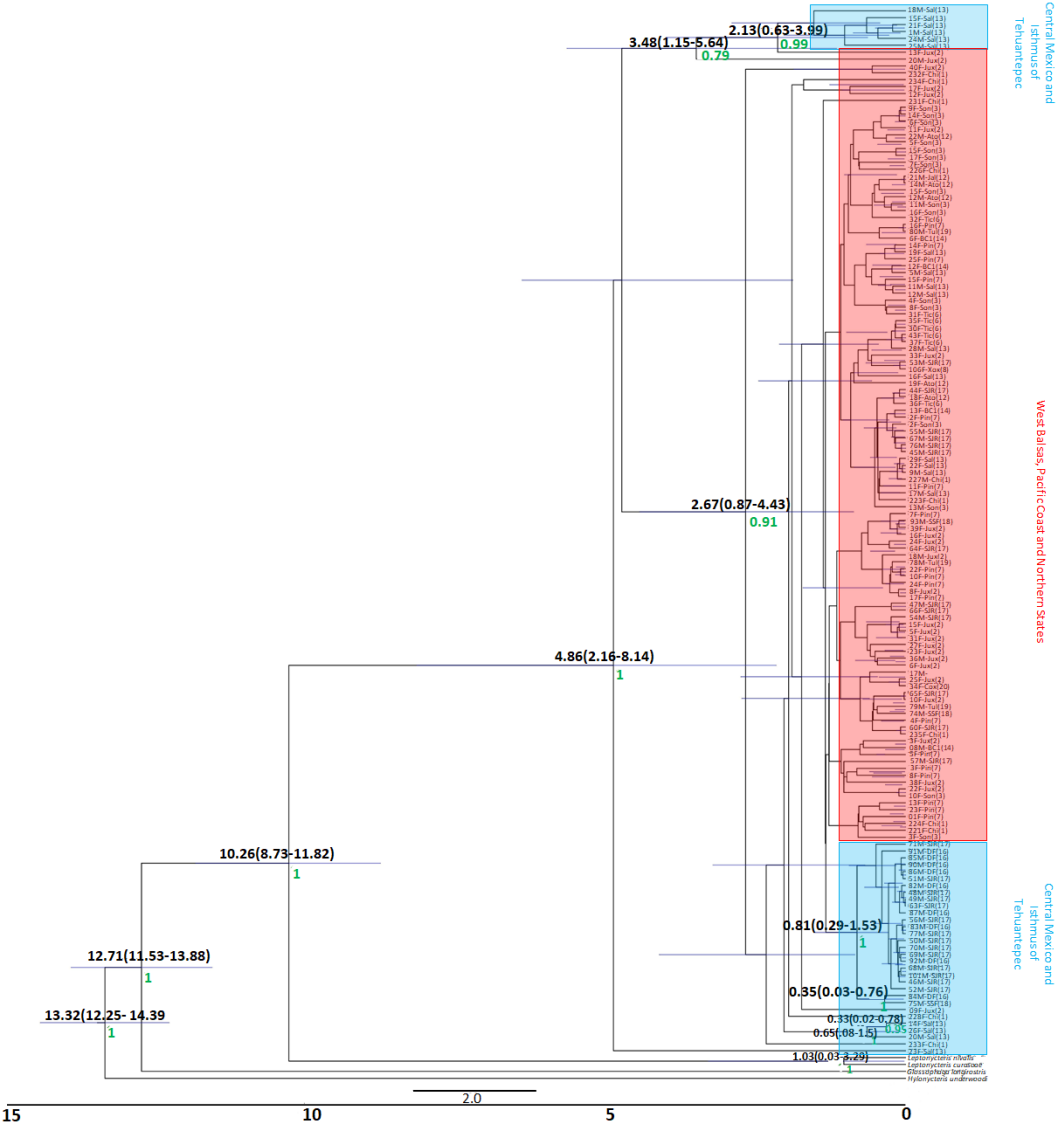
*D-loop* Gene genealogy. Gene genealogy and dates of divergence estimated with BEAST based on paternally inherited *D-loop* sequences. Black numbers above nodes depict divergence in million years; green numbers below nodes depict support values (posterior probability). Highlighted in blue color are the individuals from the Central Mexico and Isthmus of Tehuantepec localities, highlighted in red color are the individuals from the localities located in the West Balsas-Pacific Coast and Northern States Region. HDP values are in parentheses above the blue bar.

**Figure 6:**
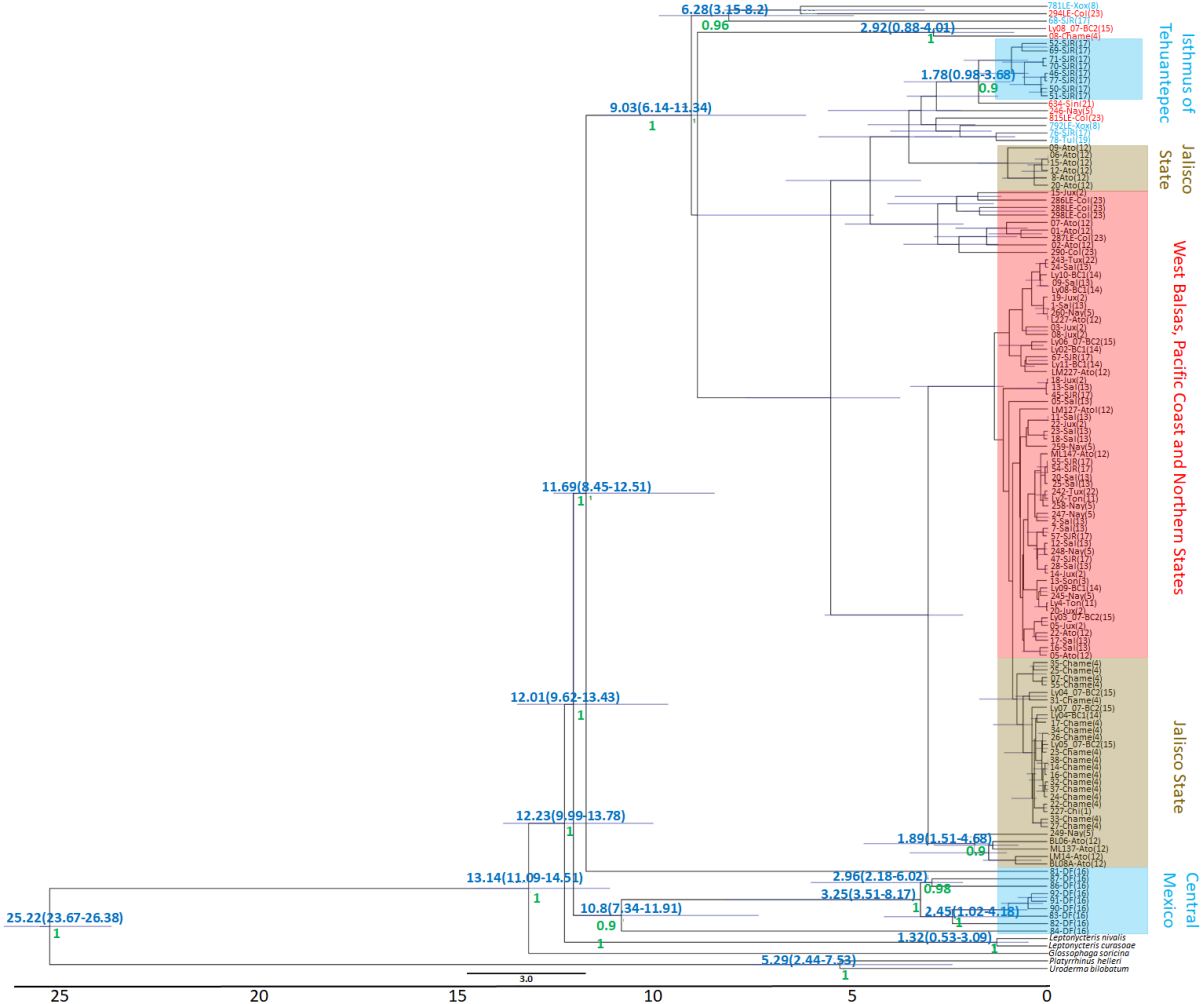
*DBY* Gene Genealogy. Gene genealogy and dates of divergence estimated with BEAST based on paternally inherited *DBY* sequences. Blue numbers above nodes depict divergence in million years; green numbers below nodes depict support values (posterior probability). Highlighted in blue color are the individuals from the Central Mexico and Isthmus of Tehuantepec localities, highlighted in red color are the individuals from the localities located in the West Balsas-Pacific Coast and Northern States Region and highlighted in brown individuals from Jalisco State . HDP values are in parentheses above the blue bar.

The Bayesian genealogy for *Cyt-b* (Fig. 4) and *D-loop* (Fig. 5) show that *L. yerbabuenae* is a monophyletic clade consisting of several lineages. The chronogram of the most probable tree constructed with mitochondrial *Cyt-b* (Fig. 4) indicated that *L. yerbabuenae* originated 4.03 mya (95% HDP, 2.27–8.63 mya). In contrast, for mitochondrial *D-loop* (Fig. 5), we obtained a deeper date of 10.26 mya (95% HDP, 8.73–11.82 mya). The chronogram based on chromosome Y *DBY* gene (Fig. 6) suggests a similar (but not overlapping, according to the 95% HDPs) date for the origin of *L. yerbabuenae*, 12.23 mya (95% HDP, 9.99–13.78 mya).

Regarding the geographical distribution of maternal lineages, for *Cyt-b*, individuals from SJR, DF and SSF are paraphyletic and show long branches denoting a possible bottleneck, and the rest of the individuals form a monophyletic clade with short branches denoting a possible population expansion (Fig. 4). For *D-loop*, there is no clear geographic structure, but we can also see that some groups have longer branches suggesting that different lineages might have undergone different demographic trajectories (Fig. 5). For the paternal lineages, the genealogy for the *DBY* gene shows several groups with a few geographical correlations among haplotypes (Fig. 6). The main group contains haplotypes from States of Pacific coast of Mexico and some members from Oaxaca and Morelos (3.06 mya; West Balsas, Pacific Coast and Northern States, Fig. 6). Regarding individuals from Central Mexico, we can see that most individuals from DF form a monophyletic clade with long branches (3.25 mya; Fig. 6), and most individuals from SJR in Oaxaca, form another clade (0.96 mya; Fig. 6). There is a group exclusively composed by members from Jalisco (1.05 mya; Fig. 6).

### Historical demography analysis

**Figure 7:**
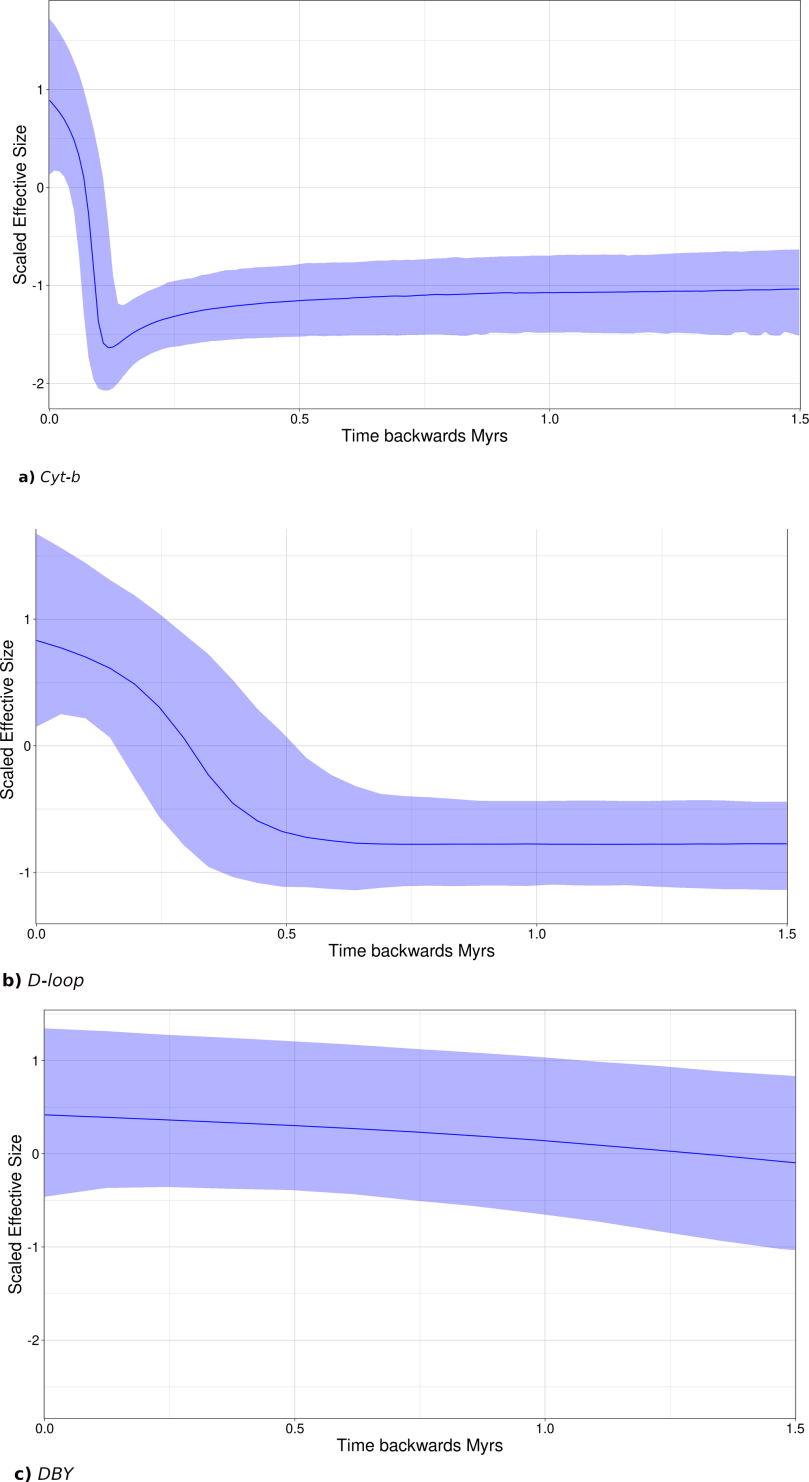
Sky-Line Plots depicting the demographic trajectory of *Leptonycteris yerbabuenae* obtained with BEAST for (A) *Cyt-b*, (B) *D-loop*, and (C) *DBY*. Results show population expansion for maternal lineages starting around the Last Interglacial Period , ∼130,000 in (A) and ∼500,000 in (B), while the paternal lineage shows demographic stability.

Bayesian skyline plots (SLP) indicate a late Pleistocene demographic expansion, starting at ∼130,000 ya for *Cyt-b* (Fig. 7A) and ∼500,000 ya for *D-loop* (Fig. 7B). In contrast, for the *DBY* gene (Fig. 7C), SLP basically exhibits demographic stability, with an older and constant but slight demographic expansion, about 6-7 mya, coincident with its own evolutionary history and divergence times dated in the gene genealogy above.

Tajima’s *D* was negative and significant for both mitochondrial genes (*Cyt-b* Tajima’s D = −1.73, *p* = 0.007; *D-loop* Tajima’s *D* =  − 1.6, *p* = 0.023; Table S4), suggesting a possible historical population expansion, while Tajima’s *D* for the *DBY* was positive and non-significant (Tajima’s *D* = 4.49, *p* = 0.99; Table S4), suggesting demographic stability at deeper times. Fu’s neutrality tests exhibited negative but non-significant values for the mitochondrial regions (*Cyt-b* = −5.86, *p* = 0.19; *D-loop* = −2.05, *p* = 0.37799 and was positive but also not significant for *DBY* Fu’s = 0.44, *p* = 0.87).

### Past distribution models

**Figure 8:**
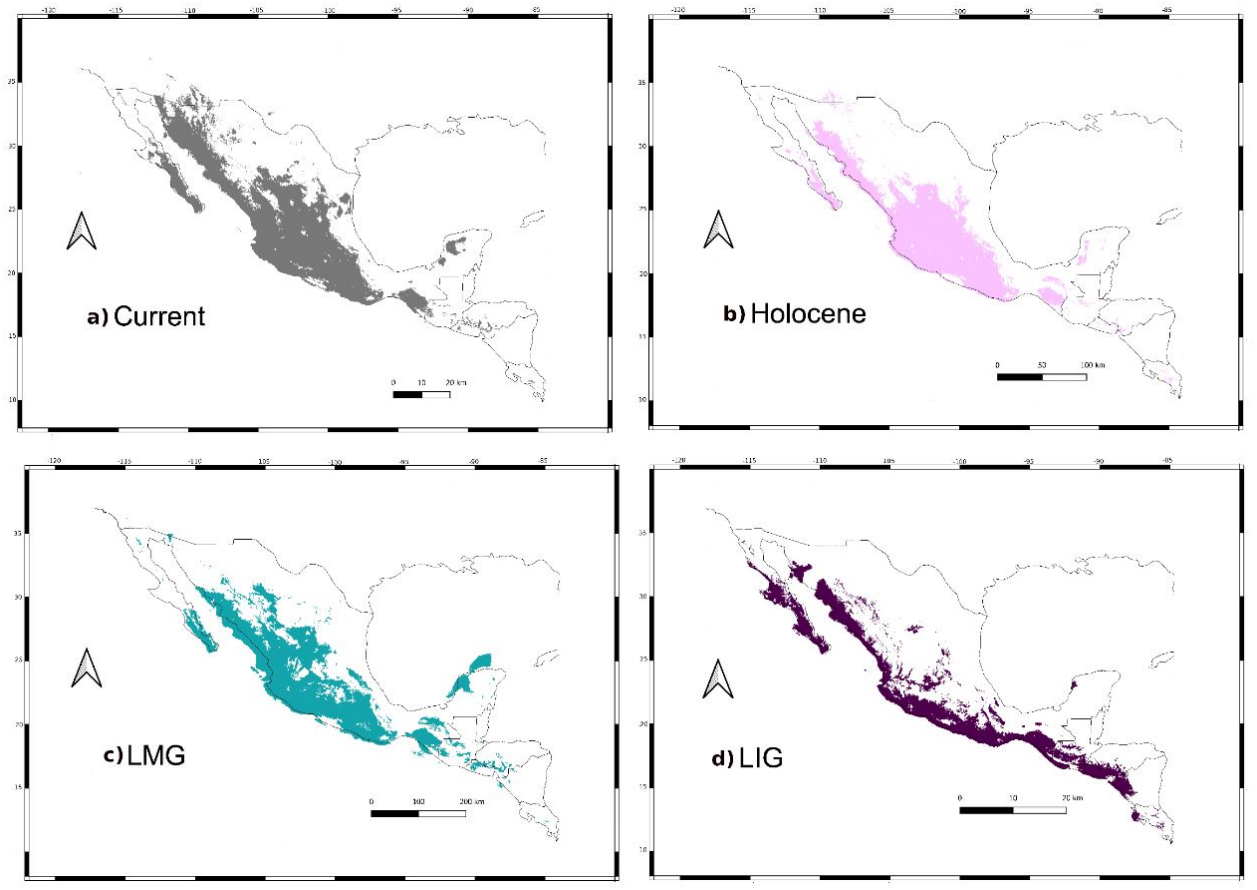
Past distribution models. Maps depict (A) Current, (B) Holocene, (C) last maximum glacial and (D) last Interglacial distribution models of *Leptonycteris yerbabuenae*.

We retained seven bioclimatic layers (Isothermality, Min Temperature of Coldest Month, Temperature Annual Range, Mean Temperature of Coldest Quarter, Precipitation of Wettest Quarter, Precipitation of Warmest Quarter, Precipitation of Coldest Quarter) to build distribution models. The potential distribution models for *L. yerbabuenae* (Figs. 2; 8) showed stability and good support, for each distribution model the area under the ROC curve (AUC) was >0.8. The projection to the Holocene (Fig. 8B) suggests that the distribution area of species has been stable during this period. Nevertheless, LIG (Fig. 8D) and LGM (Fig. 8C) models do not recover suitable environmental conditions for the presence of the species at higher latitudes, such as Arizona, USA. However, environmental suitability is predicted in West and East Balsas and the Isthmus of Tehuantepec for all periods (Fig. 2).

For the current model, the area with the greatest environmental suitability for the presence of the species is located close to West Balsas (along Sierra Madre Occidental), south of the peninsula of Baja California, the states of Oaxaca and Chiapas on the Isthmus of Tehuantepec zones (coast of the Pacific Ocean states and portions of Central Mexico; Fig. 8A). For the Holocene, *L. yerbabuenae* had a marked contraction specially in Central America, in contrast with the expansion observed in Central Mexico, West Balsas (and Pacific Coast of Mexico) and south of Baja California Peninsula (Fig. 8B). During the LGM, suitable environmental conditions occurred along the Pacific coast including West-Balsas to East-Balsas comprising areas of Central Mexico towards the southern area of the Chihuahuan Desert (Fig. 8C). The distribution of *L. yerbabuenae* apparently contracted during the LIG period but exhibited suitable conditions for the presence of species in West Balsas and the Isthmus of Tehuantepec. Finally, during the transition between the Holocene and Current periods there is an evident expansion towards the north, particularly in Sonora and Arizona (Figs. 8A–8B).

## Discussion

This study includes the largest number of samples and localities analyzed so far for *L. yebabuenae* (Wilkinson & Fleming, 1996; Morales-Garza et al., 2007; Arteaga et al., 2018; Menchaca et al., 2020). As amplification of the *D-loop* showed difficulties –apparently because of poorer preservation state of the oldest samples or a bias originated by the oligonucleotide used for its amplification such as mutations in primer binding site –we had sample size differences between molecular markers; thus, some inferences may be interpreted with caution (Jennions & Møller, 2002; Nazareno & Jump, 2012). In addition, for other migratory species, haplotype diversity is also high, for instance: *Lasiurus borealis*, *L. cinereus* and *Lasionycteris noctivagans* (Korstian JM & Williams, 2015; Sovic, Carstens & Gibbs, 2016; Vonhoff & Russell, 2015). Moreover, species where females move long distances, sex ratio bias is present and should be considered in the analyses. Therefore, we handled parental lineages separately to compare historical demographic patterns and historical distribution (Prugnolle & De Meeus, 2002). In contrast, some non-migratory species exhibit low haplotype diversity as *Dasypterus ega* and *D. intermedia* (0.018 and 0.588 respectively; Chipps et al., 2020). We found higher levels of genetic diversity, signals of stronger genetic structure and a nearly constant demographic trajectory for paternal lineages, in contrast with maternal lineages that show lower genetic variation, weak genetic structure and signals of demographic expansion.

### Genetic diversity

Levels of genetic diversity as measured by haplotype diversity for the three molecular markers (Table 2) were consistent with previous studies of this species (Wilkinson & Fleming, 1996; Morales-Garza et al., 2007; Arteaga et al., 2018; Menchaca et al., 2020), and of other American bat species (*i.e., Tadarida brasiliense* (Russell, Medellín & McCracken, 2005), *Sturnira parvidens* (Hernández-Canchola & León-Paniagua, 2017), and lower than values reported for *Artibeus jamaicensis* (Ruiz, Vargas-Miranda & Zúñiga, 2013).

The highest haplotype diversity for both mitochondrial markers was found in the Central Mexico near the Isthmus of Tehuantepec. *DBY* showed the highest genetic diversity in populations from the Pacific Coast (including Baja California Sur = 0.90; Juxtlahuaca, from Guerrero = 0.94 and Arandas, Jalisco = 0.9789) (Table S5). The conflicting patterns of genetic diversity between *DBY* and mitochondrial markers could be related to different possible causes, including their mode of inheritance, patterns of molecular evolution and distinct evolutionary and demographic history (Tosi, Morales & Melnick, 2000), and to *L. yerbabuenae*’s female migratory behavior and male philopatry (Herrera, 1997; Rojas-Martínez et al., 1999); there is a sex bias during spring-summer seasons, females are more abundant at northern latitudes when they live in roosting caves and males leave mating caves at the southern area of their distribution (Herrera, 1997; Rojas-Martínez et al., 1999; Medellín et al., 2018).

### Genetic structure

Analysis of genetic differentiation, haplotype network, BAPS and AMOVA for mtDNA showed lower genetic structure for female inherited genetic markers than for the male inherited marker, coherent with reports in other bat species (*i.e., Tadarida brasiliensis* (Russell, Medellín & McCracken, 2005), *Miniopterus schreibersii* (Bilgin et al., 2008b), *Myotis capaccinii* (Bilgin et al., 2008a) *Myotis nattereri* (Rivers, Butlin & Altringham, 2005)). In particular, for the *DBY* gene, the analyses indicate strong genetic structure with geographical consistency, comprising three groups: West of Balsas, Central Mexico-Balsas, and Isthmus of Tehuantepec (Fig. 1).

Our results support the previous inferences that *L. yerbabuenae* females move more than males, and that males show philopatric behavior, conducting only local and altitudinal movements (Cockrum, 1991; Herrera, 1997; Rojas-Martínez et al., 1999; Tellez et al., 2000; Medellín et al., 2018). Migratory females apparently promote gene flow among caves minimizing genetic differentiation, while male philopatry and perennial presence promote genetic differentiation in the *DYB* gene.

### Historical analyses

The relationships among haplotypes within *L. yerbabuenae* clade were different in the three gene genealogies; these differences were marked between the paternally (*DBY*) and one of the maternally (*Cyt-b*) inherited molecular markers, although there are some similarities. Genealogical relationship among *L. yerbabuenae* and sister species is not clear, with *D-loop* and *DBY* markers of *L. curasoae* and *L. nivalis* forming a monophyletic group. While in the case of the genealogy based on *Cyt-b*, *L. nivalis* and *L. curasoae* form independent branches, and *L. nivalis* is established as the closest species to *L. yerbabuenae* clade. In addition, we observe some disagreement in haplotype relationships depicted in the maternally inherited *Cyt-b* and *D-Loop* genealogies. In this sense, high homoplasy has been reported for the *D-loop* in several species (*Martes pennanti* in Finnilä, Lehtonen & Majamaa, 2001; *Homo sapiens* in Knaus et al., 2011).

The major difference occurred with calculated divergence times, for *DBY* marker, divergence times shows 12.01 mya [9.62–13.43 95% HDP] for the origin of *L. yerbabuenae*. These results reflect the evolutionary history for each gene, in particular differences in mutation rates (Allio et al., 2017), as well differences in the evolutionary and ecological history of each sex. Some authors have stablished that Y-chromosome genes are less polymorphic than mitochondrial genes and this could be a reason for discordant divergences times (Boissinot & Boursot, 1997).

Divergence times of *Leptonycteris yerbabuenae* for *D-loop* (10.26 mya [95% HDP, 8.73–11.82 mya]) and the chromosome Y associated *DBY* marker (12.23 mya [95% HDP, 9.99–13.78 mya]) were older than those obtained for *Cyt-b* (4.03 mya [95% HDP, 2.27−8.63 mya]). Dates of divergence for *DBY* and *D-loop* are consistent with the origin of ecologically related taxa, such as *Agave sensu lato* (4.6–12.3 mya; Flores-Abreu et al., 2019; and more recently Jiménez-Barrón et al., 2020 reported 9 mya for this group). It is interesting to note that date of divergence for *Cyt-b* is consistent with the divergence of *A. lechuguilla* (2.47–6.71 mya; (Scheinvar et al., 2017). This is relevant because *Agave* and *Leptonycteris,* are closely associated and they share close evolutionary trajectories (Rocha, Valera & Eguiarte, 2005). Times of divergence are consistent with two pulses of acceleration in the diversification rate of *Agave sensu lato*, first 8-6 mya, and second 3−2.5 mya (Good-Avila et al., 2006) and with the most recent report with a pulse at 6.18 mya and the second one at 4.91 mya (Jiménez-Barrón et al., 2020). Moreover, these dates of divergence also coincide with a temperature decrease for tropical wet climates in Mexico during glacial periods between 5.3 and 1.8 Mya (Van Devender, 2000). It is worth mentioning that in all genealogies *L. yerbabuenae* consists of several lineages that may have originated by the isolation of populations during the Pliocene, in particular in the area of Central Mexico. Nevertheless, an analysis of dates of divergence based on genome wide data will allow a better understanding of the processes leading to lineage divergence within *Leptonycteris yerbabuenae*.

The demographic history of populations also influences the shape of ultrametric trees, where a genetic bottleneck can increase the rate of coalescence of lineages, while a demographic expansion could produce isolated long branches (Ho & Shapiro, 2011; Gattepaille, Jakobsson & Blum, 2013). Accordingly, during the Pleistocene, the species may have undergone geographic expansion that erased geographic structure. In particular, one shows (*Cyt-b*) signals of demographic expansion, probably associated with Pleistocene climate changes as supported by demographic analyses.

High haplotype diversity and medium-low nucleotide diversity, in addition to a star-like haplotype network, further suggests population expansion (Slatkin & Hudson, 1991; Avise, 2000). SkyLine Plots for mtDNA suggest that a population expansion began approximately 130,000 and 500,000 years ago, during the Pleistocene; Tajima’s *D* results also support demographic expansion for mtDNA. These results are further supported by past distribution models showing an expansion from the LIG through the Holocene.

Pleistocene climate changes influenced the distribution of a variety of living groups. Overall, highland biota was fragmented during warmer interglacial periods (McDonald, 1993; Metcalfe et al., 2000; León-Paniagua et al., 2007; Ruiz et al., 2010; Bryson et al., 2011; Gugger et al., 2011), followed by expansions related to an increase in temperature (Hundertmark et al., 2002; Hofreiter & Stewart, 2009; De Bruyn, Hoelzel AR & Hofreiter, 2011). The transition between the LIG and Holocene distribution models are consistent with warmer conditions in Mexico 15,000–12,000 years ago, and with the colonization of ecologically related groups such as Cactacea and *Agave* towards the north of Mexico (Metcalfe et al., 2000). Furthermore, current distribution for *L. yerbabuenae* agrees with the distribution of several species of the genus *Agave* (Scheinvar et al., 2017; Scheinvar, 2018).

Female migration in *L. yerbabuenae* might have occurred following the changing climatic conditions of the Pleistocene. Our models for LIG, Holocene and current periods suggest an expansion to northern areas along the Pacific Coast towards Sonora. This area is home to numerous maternity colonies of *L. yerbabuenae*, including the important Pinacate roost cave (Medellín et al., 2018). The volcanic record indicates that the most recent volcanic activity in the area occurred 12,000 (+/- 4,000) years ago, while the origin of the current ecosystem has been dated 9,000 years ago (Marshall & Blake, 2009). These dates for geological and biological events coincide with our estimated expansion model for the Holocene. These models are also supported by Arteaga et al. (2018), who reported a local demographic expansion for *L. yerbabuenae* in northwestern Mexico associated to Pleistocene climate changes.

Demographic analyses among genetic markers are contrasting, suggesting a demographic expansion for the female inherited markers, but not in the male lineages. This recent expansion is further supported by past projection models. Moreover, molecular data and past distribution models suggest that *L. yerbabuenae* female’s migration to northern maternal roost has been an ecological and dynamic process originated in the Pacific Coast zone of Mexico and Central-South area (Isthmus of Tehuantepec; Figs. 8B–8D). This hypothesis is reinforced because the Pinacate area became ideal for the arrival of females only ∼8000–16,000 years ago, due in part to more favorable climatic and geologic conditions for the bat species during the climatic transition from LIG-Holocene. Moreover, genetic diversity, Bayesian genealogies for the three markers and the model for more stable thermal conditions from the Pleistocene to Current time, provide information to assume that the site of origin of the species could have been located in the Balsas zone of Mexico and Central-South (Isthmus of Tehuantepec; Fig. 1). According to the basal lineage in the gene genealogy, an ancestor of the living populations of *L. yerbabuenae* existed south of its current distribution. This origin is also supported by the greatest diversity of lineages currently present in the zone of the Isthmus of Tehuantepec. Nevertheless, these biogeographic hypotheses should be tested with other type of molecular markers, such as SNPs, with higher phylogenetic resolution and including the three species of the genus *Leptonycteris*.

## Conclusions

*Leptonycteris yerbabuenae* is rich in genetic variation, both in the mitochondria and in the DBY gene. The genetic structure suggests that migrant females promote gene flow, maintaining a cohesive species; however, male philopatry promoted genetic differentiation of three different geographic groups –West of Balsas, Central Mexico, and Isthmus of Tehuantepec. Results from demographic analyses are contrasting and suggest demographic expansion for female inherited molecular markers, but not so for male inherited DBY. This expansion is further supported by past projection models.

Information generated in this work can contribute to reinforce conservation and management strategies for *L. yerbabuenae*. Based on highest haplotype diversity sites, it is possible to design corridors in order to keep connectivity among these sites and it is of relevant importance to conservation to allow *L. yerbabuenae* to play its role as main pollinator of genus *Agave* in wild and cultivated species (Molina-Freaner & Eguiarte, 2003; Scott, 2004; Rocha et al., 2006; Trejo-Salazar et al., 2016). Thus, it is evident that ecological and evolutionary interactions among lesser long-nosed bat and *Agave* is a key for the maintenance of the major vegetation types in Mexico (dry-forest and xerophytic vegetations). Furthermore, this result can overlap with current conservation programs, as “Batfriendly” which consists of promoting the conservation of agaves grown in strategic ecological and economic areas to support the migratory movements of bats (Trejo-Salazar et al., 2016).

## Supplemental Information

10.7717/peerj.12168/supp-1 Supplemental Information 1 Pairwise *F*_*S*_*T* among *Leptonycteris yerbabuenae* populations based on *Cyt-b* marker *Negative values were interpreted as 0 (Excoffier & Lischer, 2010).

10.7717/peerj.12168/supp-2 Supplemental Information 2 Pairwise *F*_*S*_*T* among *Leptonycteris yerbabuenae* populations based on *D-loop* marker *Negative values were interpreted as 0 (Excoffier & Lischer, 2010).

10.7717/peerj.12168/supp-3 Supplemental Information 3 Pairwise *F*_*S*_*T* among *Leptonycteris yerbabuenae* populations based on *DBY* marker *Negative values were interpreted as 0 (Excoffier & Lischer, 2010).

10.7717/peerj.12168/supp-4 Supplemental Information 4 Neutrality tests, Tajima’s *D* and Fu’s *F* calculated for *Cyt-b*, *D-loop* and *DBY* regions for *Leptonycteris yerbabuenae*. Boldface numbers indicate statistic values * Indicates statistically significant values (p<0.05).

10.7717/peerj.12168/supp-5 Supplemental Information 5 Haplotype diversity calculated for locality (*Cyt-b*,*D-loop* and *DBY*) of *Leptonycteris yerbabuenae.* * Values calculated from a sample smaller than three individuals.

10.7717/peerj.12168/supp-6 Supplemental Information 6 Accession number of haplotypes Personal ID, Genbank ID and GenBank accession number for sequences used in analyses

10.7717/peerj.12168/supp-7 Supplemental Information 7 Geographical coordinates, locations information and institutional collection of records used for building Climatic Niche Models for *Leptonycteris yerbabuenae*

10.7717/peerj.12168/supp-8 Supplemental Information 8 Aligned Fasta File: Cytochrome-b Each sequence represents an individual tissue sample

10.7717/peerj.12168/supp-9 Supplemental Information 9 Aligned Fasta File: *DBY* marker Each sequence represents an individual tissue sample

10.7717/peerj.12168/supp-10 Supplemental Information 10 Aligned Fasta File: Control region (D-loop) Each sequence represents an individual tissue sample

## Additional Information and Declarations

### Field Study Permissions

The following information was supplied relating to field study approvals (i.e., approving body and any reference numbers): SEMARNAT SGPA/DGVS/07161/15.

### DNA Deposition

The following information was supplied regarding the deposition of DNA sequences: The *D-loop* sequences are available at Genbank: MT790834–MT790986; the *Cytb* sequences are available at Genbank: MT859334 to MT859403 and; the *DBY* sequences are available at Genbank: MT913638 to MT913767.

### Data Availability

The following information was supplied regarding data availability: The aligned FASTA files are available as Supplemental Files.
