# Supplementary material for: Discordance in maternal and paternal genetic markers in lesser long-nosed bat Leptonycteris yerbabuenae, a migratory bat: recent expansion to the North and male phylopatry
Source: PeerJ. 2021 Sep 29;9:e12168. doi: 10.7717/peerj.12168 (PMC8487242; doi:10.7717/peerj.12168)
Supplement: Supplemental Information 1 — *Negative values were interpreted as 0 (Excoffier & Lischer, 2010). [file peerj-09-12168-s001.docx]

**Supplemental Table S.1.**

**Discordance in maternal and paternal genetic makers in lesser long-nosed bat *Leptonycteris yerbabuenae*, a migratory bat: Recent expansion to the North and male phylopatry**

Roberto-Emiliano Trejo-Salazar^1,2*^, Gabriela Castellanos-Morales^3^, Dulce Carolina Hernández-Rosales^2^, Niza Gámez^4^, Jaime Gasca^2^, Miguel Morales^5^, Rodrigo A. Medellín^6^, Luis E. Eguiarte^2*^

**Table S.1.** Pairwise *F_ST_* among *Leptonycteris yerbabuenae* populations based on *Cyt-b* marker.

|  | Tonatico | Baja_Cal1 | Baja_Cal2 | Juxtla | Xoxafi | Pinacate | Salitre | Chamela | Chiapas | Jalisco | Nayarit | DF | Coxcatlan | Puebla | SJR | S Sebas | Tula | Hermosillo | Ticuman |
| --- | --- | --- | --- | --- | --- | --- | --- | --- | --- | --- | --- | --- | --- | --- | --- | --- | --- | --- | --- |
| Tonatico | 0 |  |  |  |  |  |  |  |  |  |  |  |  |  |  |  |  |  |  |
| Baja_Cal1 | 0.0323 | 0 |  |  |  |  |  |  |  |  |  |  |  |  |  |  |  |  |  |
| Baja_Cal2 | -0.0361 | -0.0385 | 0 |  |  |  |  |  |  |  |  |  |  |  |  |  |  |  |  |
| Juxtla | -0.0535 | 0.0455 | 0.0085 | 0 |  |  |  |  |  |  |  |  |  |  |  |  |  |  |  |
| Xoxafi | 0.1667 | 0.5833 | 0.4167 | 0.0652 | 0 |  |  |  |  |  |  |  |  |  |  |  |  |  |  |
| Pinacate | -0.0127 | -0.0216 | -0.0404 | 0.0277 | 0.4158 | 0 |  |  |  |  |  |  |  |  |  |  |  |  |  |
| Salitre | -0.0331 | 0.0308 | 0.0037 | 0.0235 | 0.3139 | 0.0217 | 0 |  |  |  |  |  |  |  |  |  |  |  |  |
| Chamela | -0.0067 | 0.0157 | -0.0060 | -0.0206 | 0.2167 | 0.0115 | 0.0332 | 0 |  |  |  |  |  |  |  |  |  |  |  |
| Chiapas | -0.0082 | -0.0386 | -0.0405 | 0.0274 | 0.4615 | -0.0216 | 0.0094 | 0.0060 | 0 |  |  |  |  |  |  |  |  |  |  |
| Jalisco | -0.0748 | -0.0090 | -0.0450 | -0.0158 | 0.2857 | -0.0240 | -0.0093 | -0.0098 | -0.0273 | 0 |  |  |  |  |  |  |  |  |  |
| Nayarit | 0.1667 | 0.5833 | 0.4167 | 0.2210 | 1.0000 | 0.4158 | 0.3139 | 0.3974 | 0.4615 | 0.2857 | 0 |  |  |  |  |  |  |  |  |
| DF | 0.0240 | 0.2248 | 0.1496 | 0.0809 | 0 | 0.1780 | 0.1305 | 0.1568 | 0.1862 | 0.0851 | 0 | 0 |  |  |  |  |  |  |  |
| Coxcatlan | -0.6667 | -0.8750 | -0.7500 | -0.4381 | 1.0000 | -0.6692 | -0.4867 | -0.5667 | -0.7500 | -0.6667 | 1.0000 | -0.1000 | 0 |  |  |  |  |  |  |
| Puebla | -0.0871 | 0.0246 | -0.0286 | -0.0226 | 0.2000 | -0.0064 | -0.0256 | -0.0192 | -0.0263 | -0.0587 | 0.2000 | 0.0474 | -0.6000 | 0 |  |  |  |  |  |
| SJR | 0.0036 | 0.1336 | 0.0859 | 0.0474 | 0.1118 | 0.1044 | 0.0829 | 0.0838 | 0.1058 | 0.0482 | 0.1673 | 0.0353 | -0.2111 | 0.0235 | 0 |  |  |  |  |
| San_Seb | 0.0780 | 0.3435 | 0.2403 | 0.1368 | 0 | 0.2681 | 0.2007 | 0.2425 | 0.2853 | 0.1549 | 0 | 0 | 0 | 0.1045 | 0.1034 | 0 |  |  |  |
| Tula | 0.0769 | -0.1250 | -0.0678 | 0.0453 | 1.0000 | -0.0577 | 0.0209 | 0.0011 | -0.0833 | -0.0096 | 1.0000 | 0.2500 | 0 | 0.0400 | 0.1533 | 0.3878 | 0 |  |  |
| Hermosillo | -0.0560 | 0.0588 | 0.0045 | 0.0018 | 0.1373 | 0.0295 | 0.0228 | 0.0322 | 0.0352 | -0.0217 | 0.1373 | 0.0377 | -0.4118 | -0.0360 | 0.0356 | 0.0816 | 0.0649 | 0 |  |
| Ticuman | -0.0543 | -0.0182 | -0.0424 | -0.0016 | 0.3333 | -0.0223 | -0.0292 | -0.0049 | -0.0284 | -0.0493 | 0.3333 | 0.1160 | -0.6667 | -0.0417 | 0.0669 | 0.1936 | -0.0345 | -0.0032 | 0 |

*Negative values were interpreted as 0 (Excoffier and Lischer, 2010).
