# Supplementary material for: Discordance in maternal and paternal genetic markers in lesser long-nosed bat Leptonycteris yerbabuenae, a migratory bat: recent expansion to the North and male phylopatry
Source: PeerJ. 2021 Sep 29;9:e12168. doi: 10.7717/peerj.12168 (PMC8487242; doi:10.7717/peerj.12168)
Supplement: Supplemental Information 2 — *Negative values were interpreted as 0 (Excoffier & Lischer, 2010). [file peerj-09-12168-s002.docx]

**Supplemental Table S.2.**

**Discordance in maternal and paternal genetic makers in lesser long-nosed bat *Leptonycteris yerbabuenae*, a migratory bat: Recent expansion to the North and male phylopatry**

Roberto-Emiliano Trejo-Salazar^1,2*^, Gabriela Castellanos-Morales^3^, Dulce Carolina Hernández-Rosales^2^, Niza Gámez^4^, Jaime Gasca^2^, Miguel Morales^5^, Rodrigo A. Medellín^6^, Luis E. Eguiarte^2*^

**Table S.2.** Pairwise *F_ST_* among *Leptonycteris yerbabuenae* populations based on *D-loop* marker.

|  | Baja_Cal1 | Salitre | Juxtla | SJR | San_Seb | Tula | DF | Pinacate | Hermosillo | Chiapas | Coxcatlan | Chamela | Jalisco | Xoxafi | Ticuman |
| --- | --- | --- | --- | --- | --- | --- | --- | --- | --- | --- | --- | --- | --- | --- | --- |
| Baja_Cal1 | 0 |  |  |  |  |  |  |  |  |  |  |  |  |  |  |
| Salitre | 0.0572 | 0 |  |  |  |  |  |  |  |  |  |  |  |  |  |
| Juxtla | -0.0153 | 0.1106 | 0 |  |  |  |  |  |  |  |  |  |  |  |  |
| S J R | 0.0162 | 0.1866 | 0.0818 | 0 |  |  |  |  |  |  |  |  |  |  |  |
| San_Seb | 0.0743 | 0.0276 | 0.0597 | 0.1652 | 0 |  |  |  |  |  |  |  |  |  |  |
| Tula | -0.0909 | 0.0360 | -0.0849 | 0.0110 | 0.0000 | 0 |  |  |  |  |  |  |  |  |  |
| DF | 0.2280 | 0.2057 | 0.2589 | 0.1279 | 0.1771 | 0.2084 | 0 |  |  |  |  |  |  |  |  |
| Pinacate | -0.0833 | 0.1607 | 0.0412 | 0.0742 | 0.2013 | -0.1143 | 0.3301 | 0 |  |  |  |  |  |  |  |
| Hermosillo | -0.0540 | 0.1976 | 0.0618 | 0.0847 | 0.2741 | -0.1740 | 0.3297 | 0.0050 | 0 |  |  |  |  |  |  |
| Chiapas | -0.0262 | 0.0846 | 0.0238 | 0.0912 | 0.1220 | -0.0402 | 0.2659 | 0.0380 | 0.1030 | 0 |  |  |  |  |  |
| Coxcatlan | -1.0000 | -0.4659 | -0.7239 | -0.5065 | -1.0000 | 0.0000 | -0.2008 | -0.8016 | -0.9509 | -0.6691 | 0 |  |  |  |  |
| Chamela | -2.0000 | -5.4580 | -3.0540 | -4.7417 | -10 | 1.0000 | -5.6042 | -2.8216 | -2.9018 | -2.7556 | 1 | 0 |  |  |  |
| Jalisco | -0.1682 | 0.1102 | 0.0056 | 0.0470 | 0.1752 | -0.1539 | 0.2793 | -0.0571 | -0.0827 | 0.0220 | -1.0000 | -1.2500 | 0 |  |  |
| Xoxafi | 0.2800 | -0.4001 | -0.5632 | -0.3708 | -0.8049 | 1.0000 | -0.0567 | 0.0070 | 0.2278 | -0.2180 | 1.0000 | 1.0000 | 0.4194 | 0 |  |
| Ticuman | 0.1569 | 0.1135 | -0.0102 | 0.0191 | 0.1647 | 0.2028 | 0.3062 | 0.0841 | 0.1752 | 0.0861 | -0.2533 | -1.6111 | 0.1720 | -0.6491 | 0 |

*Negative values were interpreted as 0 (Excoffier and Lischer, 2010).
