# Supplementary material for: Discordance in maternal and paternal genetic markers in lesser long-nosed bat Leptonycteris yerbabuenae, a migratory bat: recent expansion to the North and male phylopatry
Source: PeerJ. 2021 Sep 29;9:e12168. doi: 10.7717/peerj.12168 (PMC8487242; doi:10.7717/peerj.12168)
Supplement: Supplemental Information 3 — *Negative values were interpreted as 0 (Excoffier & Lischer, 2010). [file peerj-09-12168-s003.docx]

**Supplemental Table S.3.**

**Discordance in maternal and paternal genetic makers in lesser long-nosed bat *Leptonycteris yerbabuenae*, a migratory bat: Recent expansion to the North and male phylopatry**

Roberto-Emiliano Trejo-Salazar^1,2*^, Gabriela Castellanos-Morales^3^, Dulce Carolina Hernández-Rosales^2^, Niza Gámez^4^, Jaime Gasca^2^, Miguel Morales^5^, Rodrigo A. Medellín^6^, Luis E. Eguiarte^2*^

**Table S.3.** Pairwise *F_ST_* among *Leptonycteris yerbabuenae* populations based on *DBY* marker.

|  | Baja_Cal1 | Colima | Nayarit | Jalisco | Arandas | Sinaloa | Xoxafi | Hermosillo | Chamela | Chiapas | DF | Juxtla | Salitre | S J R | Tula | Tonatico |
| --- | --- | --- | --- | --- | --- | --- | --- | --- | --- | --- | --- | --- | --- | --- | --- | --- |
| Baja_Cal1 | 0 |  |  |  |  |  |  |  |  |  |  |  |  |  |  |  |
| Colima | 0.2219 | 0 |  |  |  |  |  |  |  |  |  |  |  |  |  |  |
| Nayarit | 0.3249 | 0.3857 | 0 |  |  |  |  |  |  |  |  |  |  |  |  |  |
| Jalisco | -0.2916 | -0.2777 | -0.9143 | 0 |  |  |  |  |  |  |  |  |  |  |  |  |
| Arandas | 0.2190 | 0.2190 | 0.0196 | -0.8426 | 0 |  |  |  |  |  |  |  |  |  |  |  |
| Sinaloa | -0.2285 | -0.2606 | 0.5271 | 1.0000 | -0.6125 | 0 |  |  |  |  |  |  |  |  |  |  |
| Xoxafi | 0.1899 | -0.3403 | 0.6901 | -0.9546 | 0.2974 | -0.9482 | 0 |  |  |  |  |  |  |  |  |  |
| Hermosillo | -0.2889 | -0.2726 | -0.1167 | 1.0000 | -0.7598 | 1.0000 | -0.9609 | 0 |  |  |  |  |  |  |  |  |
| Chamela | 0.3137 | 0.5666 | 0.8784 | 0.8484 | 0.5441 | 0.8484 | 0.8002 | 0.8484 | 0 |  |  |  |  |  |  |  |
| Chiapas | -0.4510 | -0.0804 | 0.9242 | 1.0000 | 0.1730 | 1.0000 | -0.7054 | 1.0000 | -0.9685 | 0 |  |  |  |  |  |  |
| DF | 0.1398 | 0.3584 | 0.6585 | 0.3219 | 0.3843 | 0.3407 | 0.4854 | 0.3219 | 0.4854 | -0.1202 | 0 |  |  |  |  |  |
| Juxtla | 0.7722 | 0.6497 | 0.9819 | 0.9737 | 1 | 0.9737 | 0.7895 | 0.9737 | 0.9751 | 0.9734 | 1 | 0 |  |  |  |  |
| Salitre | 0.8274 | 0.7362 | 0.9886 | 0.9887 | 0.8827 | 0.9887 | 0.8782 | 0.9887 | 0.9816 | 0.9886 | 0.9452 | 0.1106 | 0 |  |  |  |
| S J R | 0.8129 | 0.7194 | 0.9668 | 0.9539 | 0.8704 | 0.9539 | 0.8311 | 0.9539 | 0.9649 | 0.9534 | 0.9243 | 0.2372 | 0.2715 | 0 |  |  |
| Tula | 0.6266 | 0.3425 | 0.9886 | 1.0000 | 0.7980 | 1.0000 | -0.2016 | 1.0000 | 0.9761 | 1.0000 | 0.8665 | 0.7766 | 0.8978 | 0.6449 | 0 |  |
| Tonatico | 0.67469 | 0.46748 | 0.98937 | 0.99688 | 0.81546 | 0.99688 | 0.38115 | 0.99688 | 0.97706 | 0.99685 | 0.88356 | -0.06709 | 0.09962 | 0.00822 | 0.97015 | 0 |

*Negative values were interpreted as 0 (Excoffier and Lischer, 2010).
