# Supplementary material for: Discordance in maternal and paternal genetic markers in lesser long-nosed bat Leptonycteris yerbabuenae, a migratory bat: recent expansion to the North and male phylopatry
Source: PeerJ. 2021 Sep 29;9:e12168. doi: 10.7717/peerj.12168 (PMC8487242; doi:10.7717/peerj.12168)
Supplement: Supplemental Information 4 — * Indicates statistically significant values (p<0.05). [file peerj-09-12168-s004.docx]

**Supplemental Table S.4.**

**Discordance in maternal and paternal genetic makers in lesser long-nosed bat *Leptonycteris yerbabuenae*, a migratory bat: Recent expansion to the North and male phylopatry**

Roberto-Emiliano Trejo-Salazar^1,2*^, Gabriela Castellanos-Morales^3^, Dulce Carolina Hernández-Rosales^2^, Niza Gámez^4^, Jaime Gasca^2^, Miguel Morales^5^, Rodrigo A. Medellín^6^, Luis E. Eguiarte^2*^

**Table S.4.** Results from neutrality tests, Tajima’s *D* and Fu’s *F* calculated for *Cyt-b*, *D-loop* and *DBY* regions for *Leptonycteris yerbabuenae*.

| Statistics |  | ***Cyt-b*** | ***D-loop*** | ***DBY*** |
| --- | --- | --- | --- | --- |
| Tajima's D test | | | | |
|  | Sample size | 213 | 153 | 132 |
|  | S | 311 | 168 | 408 |
|  | Pi | 23.79675 | 15.03096 | 176.50937 |
|  | Tajima's D | **-1.73241** | **-1.60453** | **4.49574** |
|  | Tajima's D p-value | **0.007*** | **0.023*** | **0.999** |
| Fu's FS test | | | | |
|  | No. of alleles | 72 | 43 | 98 |
|  | Theta_pi | 23.79675 | 15.03096 | 176.50937 |
|  | Exp. no. of alleles | 55.1299 | 36.74605 | 98.77324 |
|  | FS | **-5.86524** | **-2.0515** | **0.44674** |
|  | FS p-value | **0.19** | **0.377** | **0.879** |

* Indicates statistically significant values (p<0.05)
