# Supplementary material for: Discordance in maternal and paternal genetic markers in lesser long-nosed bat Leptonycteris yerbabuenae, a migratory bat: recent expansion to the North and male phylopatry
Source: PeerJ. 2021 Sep 29;9:e12168. doi: 10.7717/peerj.12168 (PMC8487242; doi:10.7717/peerj.12168)
Supplement: Supplemental Information 5 — * Values calculated from a sample smaller than three individuals. [file peerj-09-12168-s005.docx]

**Supplemental Table S.5.**

**Discordance in maternal and paternal genetic makers in lesser long-nosed bat *Leptonycteris yerbabuenae*, a migratory bat: Recent expansion to the North and male phylopatry**

Roberto-Emiliano Trejo-Salazar^1,2*^, Gabriela Castellanos-Morales^3^, Dulce Carolina Hernández-Rosales^2^, Niza Gámez^4^, Jaime Gasca^2^, Miguel Morales^5^, Rodrigo A. Medellín^6^, Luis E. Eguiarte^2*^

**Table S.5** Haplotype diversity obtained for each locality (Cyt-b, D-loop and DBY) of *Leptonycteris yerbabuenae*.

|  | | *Hd* | | |
| --- | --- | --- | --- | --- |
| Locality | | *Cyt-b* | *D-loop* | *DBY* |
| 1 Los Laguitos, Chiapas | Chi | 0.5385 | 0.9091 | 1* |
| 2 Juxtlahuaca, Guerrero | Jux | 0.779 | 0.78 | 0.9444 |
| 3 La Mariana, Sonora | son | 0.8627 | 0.45 | 1* |
| 4 Chamela, Jalisco | Chame | 0.6026 | 1* | 0.6725 |
| 5 Las Lumbres, Nayarit | Nay | 1* | - | 0.8182 |
| 6 Ticuman, Morelos | Tic | 0.6667 | 0.6667 | - |
| 7 El Pinacate, Sonora | Pin | 0.5842 | 0.538 | - |
| 8 Xoxafi, Hidalgo | Xox | 1* | 1* | 1* |
| 9 Las Vegas, Puebla | vegas | 0.8 | - | - |
| 10 Tzinacanostoc, Puebla | Tzni | - | - | - |
| 11 Tonatico, Estado de México | Ton | 0.8333 | - | 1* |
| 12 Atotonilco, Jalisco | Ato | 0.7143 | 0.3333 | 0.9789 |
| 13 Tetecalita, Morelos (Salitre) | Sal | 0.6862 | 0.9842 | 0.875 |
| 14 Baja California (Las Cuevas) | BC1 | 0.4167 | 0.5 | 0.9091 |
| 15 Baja California (Mulege) | BC2 | 0.5833 | - | - |
| 16 Ciudad de México | DF | 0.9 | 0.8611 | 1* |
| 17 San Juan Raya, Oaxaca | SJR | 0.8327 | 0.8205 | 1 |
| 18 San Sebastian Frontera, Oaxaca | SSF | 1 | 1 | - |
| 19 Tula, Hidalgo | Tul | 1* | 1* | 1* |
| 20 Coxcatlán, Oaxaca | Cox | 1* | 1* | - |
| 21 Navachiste, Sinaloa | Sin | - | - | 1* |
| 22 Tuxtepec, Jalisco | Tux | - | - | - |
| 23 Callejones, Colima | Col | - | - | 1 |

* Values calculated from a sample smaller than three individuals.
